# De Novo 1q21.3q22 Duplication Revaluation in a “Cold” Complex Neuropsychiatric Case with Syndromic Intellectual Disability

**DOI:** 10.3390/genes12040511

**Published:** 2021-03-31

**Authors:** Roberta Milone, Roberta Scalise, Rosa Pasquariello, Stefano Berloffa, Ivana Ricca, Roberta Battini

**Affiliations:** 1 Department of Developmental Neuroscience, IRCCS Stella Maris Foundation, Calambrone, 56128 Pisa, Italy; rmilone@fsm.unipi.it (R.M.); rscalise@fsm.unipi.it (R.S.); rpasquariello@fsm.unipi.it (R.P.); sberloffa@fsm.unipi.it (S.B.); 2 Tuscan PhD Program of Neuroscience, University of Florence, Pisa and Siena, 50139 Florence, Italy; 3 Molecular Medicine, IRCCS Stella Maris Foundation, 56128 Pisa, Italy; iricca@fsm.unipi.it; 4 Department of Clinical and Experimental Medicine, University of Pisa, 56125 Pisa, Italy

**Keywords:** spastic paraparesis, movement disorder, *UBQLN4*, *ASH1L*, *SYT11*, *RIT1*

## Abstract

Syndromic intellectual disability often obtains a genetic diagnosis due to the combination of first and next generation sequencing techniques, although their interpretation may require revaluation over the years. Here we report on a composite neuropsychiatric case whose phenotype includes moderate intellectual disability, spastic paraparesis, movement disorder, and bipolar disorder, harboring a 1.802 Mb de novo 1q21.3q22 duplication. The role of this duplication has been reconsidered in the light of negativity of many other genetic exams, and of the possible pathogenic role of many genes included in this duplication, potentially configuring a contiguous gene-duplication syndrome.

## 1. Introduction

Intellectual disability (ID) is the most common neurodevelopmental disorder, affecting 1–3% of the world population, although its frequency varies worldwide [1]. According to the Diagnostic and Statistical Manual of Mental Disorders, 5th Edition, ID is characterized by significant limitations in both intellectual functioning and adaptive behavior [2]. An intellectual quotient (IQ) below 70 indicates deficits in intellectual functioning, that combined with adaptive functioning determines further classification as mild, moderate, severe, or profound. ID has both genetic or non-genetic origin (such as maternal infections during pregnancy, exposure to toxic substances, nutritional deficiencies, hypoxic-ischemic, and traumatic injury) [3]. The association between ID and motor disorders should be considered as a hint on a syndromic condition, especially if comorbid with psychiatric disorders. Many of the syndromic conditions have monogenic origin, which is diagnosable both proceeding “gene by gene” and, more recently, through next generation sequencing [4] integrated with Sanger sequencing [5]. However, array-comparative genomic hybridization (Array-CGH) continues to be employed as a diagnostic tool in syndromic ID. Indeed, it enables to reveal genomic imbalances caused by microduplications and microdeletions that cause gene dosage alterations, with possible composite clinical pictures [6].

Here we report on a female patient with a proteiform neuropsychiatric phenotype, who underwent numerous genetic tests, and whose diagnosis has been achieved revaluating her Array-CGH analysis, in the light of a more advanced knowledge on its gene contents and function. She harbored a 1.802 Mb de novo 1q21.3q22 duplication, and showed spastic paraplegia, dyskinetic movement disorder, ID, and bipolar disorder.

Spastic paraplegia is characterized by progressive spasticity of lower limbs due to length-dependent corticospinal tract and dorsal column degeneration [7], while movement disorders reflect a dysfunction of extrapyramidal circuits due to brain lesions or to infective, toxic, autoimmune and genetic etiologies [8]. Bipolar disorder is a severe chronic mood disorder characterized by episodes of mania/hypomania with alternating or intertwining episodes of depression [9].

In contrast to the recurrent 1q21.1 copy number variant (CNV), strongly associated with ID, autism spectrum disorder, epilepsy, psychiatric diagnoses, and congenital malformations [10], distal duplications have been rarely reported in the literature, without a recognizable phenotype.

For this reason, a detailed description of clinical and neuroimaging features related to 1q21.3q22 duplication may contribute to expanding the knowledge on the gene content of this chromosomal region in the light of genotype–phenotype correlations. Furthermore, we report a 12-year follow-up of this rare condition, contributing to a better definition of its outcome and prognosis.

## 2. Material and Methods

### 2.1. Patient

The patient is a 17-year-old girl who attained her first evaluation at our institute at the age of 5. Her long clinical disease course has been followed until current age, through neurological and psychiatric evaluations, instrumental exams, and genetic analysis.

### 2.2. Molecular Analysis

G-banded karyotyping, Sanger sequencing, Array-CGH analysis, and next generation sequencing have been performed to investigate the genetic etiology of the proposita’s clinical picture.

Array-CGH analysis was performed using the Agilent 8 × 60 K microarray oligonucleotide platform with a median resolution of 100 Kbp, following manufacture’s protocol (Agilent Technologies, Santa Clara, CA, USA). The detected genomic imbalance coordinates refer to the Genome Reference Consortium Human Build 37 (GRCh37/hg19). The CNV was confirmed by quantitative polymerase chain reaction (qPCR). Segregation analyses in parental DNA were performed by qPCR. Polymorphic CNVs, based on the Database of Genomic Variants data (DGV; http://projects.tcag.ca/variation/, accessed on 16 December 2016), were filtered out. A validated multigene panel was used to identify causative mutations in 117 genes, reported in association with spastic paraplegia [11]. Gene variants were confirmed by direct Sanger sequencing.

## 3. Results 

### 3.1. Case Report

Family history was positive for bipolar disorder and anxiety in the maternal line. The proposita was the firstborn of not-consanguineous parents. Birth occurred after an uneventful pregnancy characterized by reduced fetal movements and a prolonged labor, with consequent mild perinatal suffering (Apgar scores were 6 at the first minute and 8 at the fifth). At birth, weight was 2770 g (tenth to twenty-fifth centile), length was 48 cm (twenty-fifth centile), and occipitofrontal circumference (OFC) was 32.5 cm (tenth to twenty-fifth centile). Suction was weak. Motor milestones have been acquired in delay. She acquired head control at 4 months, trunk control at 10 months, crawling at 18 months, supported ambulation with a medical walker from 30 months. She uttered her first words at the age of 3 years, and her first sentences after the age of 4.

**Figure 1:**
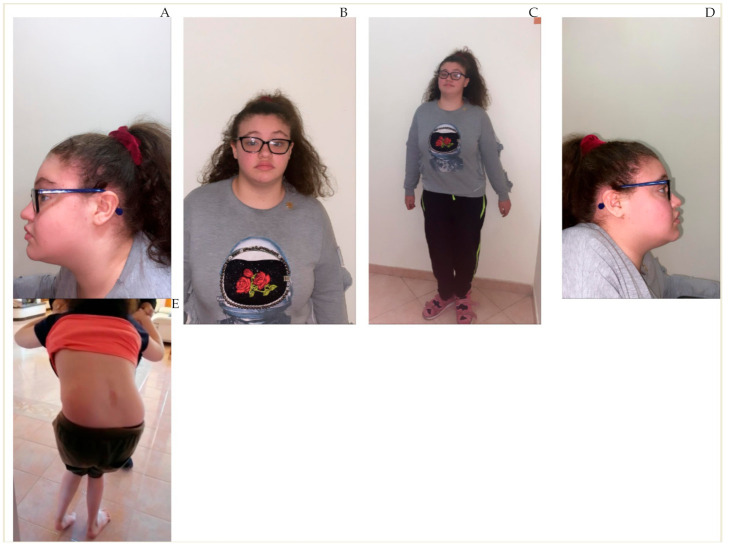
Phenotypic features and neurologic findings of the patient. Distinctive findings: Curly hair (**A**,**B**), coarse face with thickened eyebrows (**B**), ptosis (**B**,**C**), epicanthal folds (**B**), bulbous nose (**B**), flashy lips (**B**), everted upper lip (**B**), thickened helix (**A**), small low-set ears (**A–D**), short neck (**D**). Roto-scoliotic left curve at lumbar segment with right hip elevation (**E**). Lower limbs muscle hypotrophy and foot deformities (pes equinovarus) (**E**).

At the last examination, physical features included short stature (height 148 cm, −2 standard deviations) with relative macrocephaly (OFC 54.5 cm, approximately fiftieth centile), overweight (60 kg, fiftieth to seventy-fifth centile, body mass index 27.4 kg/m^2^), curly hair, coarse face with thickened eyebrows, ptosis, epicanthal folds, bulbous nose, flashy lips, everted upper lip, thickened helix, small low-set ears, and short neck (Figure 1).

Neurological examination detected dyskinetic movement disorder and spastic paraplegia, gesture dysmetria and tremors, alternating exotropia, and nystagmus. Movement disorder was not L-Dopa responsive, and did not ameliorate with anticholinergic treatment. The botulinum toxin injections in the gastrocnemius resulted in a transient decrease in spasticity at lower limbs. Antispastic therapy (Baclofen) initially per os and especially through Baclofen pump positioning reduced the hyperkinetic movement disorder, rigidity, and spasticity.

The cognitive level, evaluated with the Weschler Intelligence Scale for Children—IV edition, was referable to a moderate ID (Full Scale Intelligence Quotient 42: Verbal Comprehension Index 64, Perceptual Reasoning Index 50, Working Memory Index 52, Processing Speed Index 59).

Psychiatric evaluation diagnosed bipolar disorder, anxiety disorder, and obsessive traits. Treatment included lithium variably associated with second generation antipsychotic drugs (i.e., Quetiapine and Aripiprazole) during depressive or maniacal states, and with selective serotonin inhibitors reuptake (i.e., Fluvoxamine or Sertraline), in order to reduce obsessiveness and anxiety, conferring transient relief. Furthermore, Gabapentin showed a good efficacy both for pain and anxiety control. 

### 3.2. Instrumental Examination

Ophthalmologic examination confirmed alternating exotropia, and detected horizontal, rotatory, and vertical nystagmus. *Fundus oculi* revealed papillary pallor.

Echocardiography and electrocardiogram were normal.

Neurophysiological exams such as electroencephalogram and brainstem auditory evoked potentials were normal. Somatosensorial-evoked potentials showed significant conduction anomalies, with expression of corticospinal tract dysfunction. Motor-evoked potentials were normal. Nerve conduction velocities were also normal, while electromyography detected mild denervation signs.

Spine radiography detected wide-range left-convex lumbar rotoscoliosis and right hip elevation, associated with tibial dysmetria.

**Figure 2:**
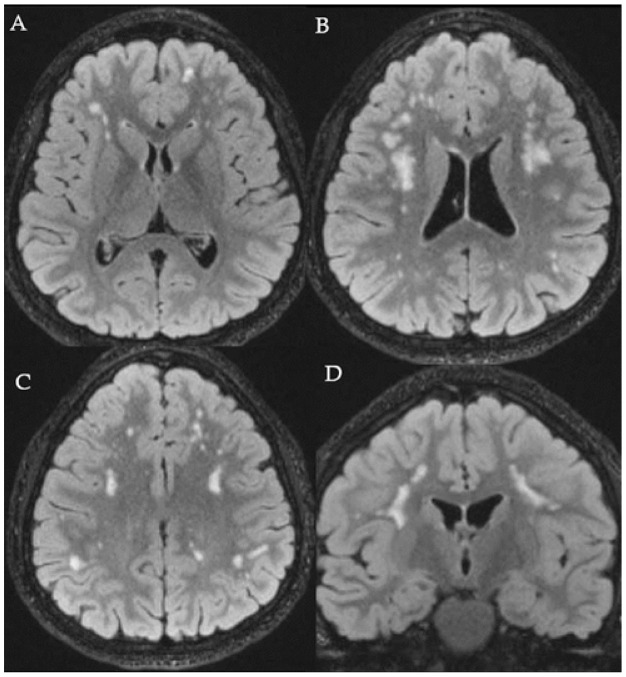
Brain MRI of the patient axial (**A**–**C**) and coronal (**D**) FLAIR images show multifocal and confluent abnormalities in the subcortical white matter of the frontoparietal lobes; stable during the follow-up.

Brain magnetic resonance imaging (MRI), performed at the age of 7, detected multifocal and partially confluent subcortical frontoparietal white matter anomalies; spinal MRI was normal; however, it incidentally revealed a bilateral horseshoe kidney. Brain MRI features have been maintained stable during the long follow-up (Figure 2).

### 3.3. Biochemical and Molecular Analysis

Numerous biochemical and metabolic investigations have been performed (i.e., blood and urine amino acids, urine organic acids, very long-chain fatty acids, phytanic acid, pristanic acid, oxysterols, sialotransferrins, alpha-fetoprotein, carcinoembryonic antigen, creatinkinases, ceruloplasmin, copper, cholesterol, biotinidase), resulting as normal. In addition, lysosomal enzyme dosage displayed mild reduction of arylsulfatase A. Cerebrospinal fluid neurotransmitter dosage (i.e., pterins, biogenic amines) was also normal.

Variants of pathogenic significance in several genes (i.e., *MECP2*, *SPG3A*, *SPG4*, *SPG7*, *SPG11*, and *DYT1*) were ruled out; karyotype analysis was normal (46, XX). 

A next generation panel including 117 genes associated with spastic paraplegia was carried out and detected two heterozygous variants of unknown significance: c.929C>T (p.310L) in *ARSI*, and c.1354G>C (p.G452R) in *ENTPD1*, both related to recessive inheritance.

**Figure 3:**
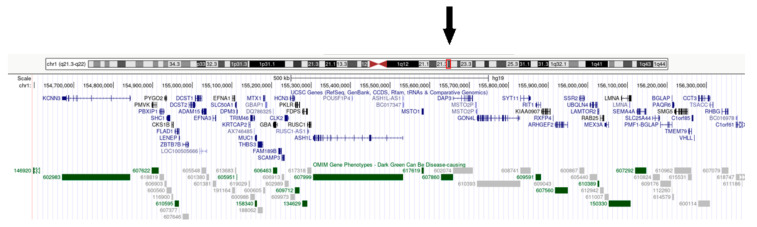
1q21.3q22 region, duplicated in our patient and its gene content, highlighted by the black arrow.

Array-CGH analysis detected a 1q21.3q22 duplication sizing about 1.802 Mb (breakpoints 154597309-156399325, GRCh37/hg19) (Figure 3). Segregation analyses in parental DNA confirmed its de novo origin.

## 4. Discussion

Anecdotal reports about 1q21.3 or 1q22 CNVs have been reported in the literature [12,13,14,15]. However, comparisons with the phenotypes previously described are difficult, since the CNVs described so far are not or only partially overlapping with ours. ID, anxiety disorder, and mood disorders have been related to 1q22 duplication [15], while 1q21.3 CNVs have been associated with hypotonia and dysmorphisms, besides ID [12,14].

To the best of our knowledge, a duplication involving both regions has never been reported. In particular, to date, such a complex neurological picture, characterized by the association of spasticity and dyskinetic movement disorder, has not been described.

1q21.3q22 region harbors several genes mainly expressed in the brain, that could potentially explain the pathology observed.

*UBQLN4* belongs to the ubiquitin-like family of proteins implied in degradative processes, especially in the ubiquitin proteasome system and autophagy. These are crucial for neuronal development (including neurite outgrowth and axon guidance), and in maintenance of homeostasis in the ageing nervous system. Therefore, *UBQLN4* dysfunction may confer vulnerability to motor neurons, making them susceptible to neurodegeneration [16]. Its overexpression might lead to cell cycle arrest and apoptosis, with dire consequences on the development, function, and survival of affected motor neurons [17]. These processes may contribute to provoke spastic paraparesis in our patient. Autophagy itself is nowadays recognized as a transversal mechanism for neurodevelopmental and neurodegenerative disorders, as spastic paraplegia, movement disorders, and ID [18].

Murine models have demonstrated that increasing *SYT11* levels are sufficient to trigger basal ganglia toxicity. Its overexpression markedly decreases the dopamine release due to the loss of dopaminergic neurons [19]. We propose *SYT11* as a candidate gene in the contribution to the dyskinetic movement disorder.

*RIT1*, involved in Noonan syndrome 8 (MIM#615355), may be related to her physical features [20], and may cause ID together with *ASH1L*.

*ASH1L* has been related to many developmental disorders and neuropsychiatric illnesses. It encodes histone H3-methyltransferases, and acts as a chromatin modifier. It may also bind to the promoter region of *NRXN1*, inhibiting its transcriptional activity, and altering synapse formation and excitatory–inhibitory balance. As an epigenetic regulator, *ASH1L* has multiple targets implied in the connectivity of neurons, and in modulating neurotrophin signaling, especially the BDNF-TrkB pathway. Therefore, *ASH1L* has pleiotropic effects, and is involved in ID, and other neurodevelopmental and psychiatric disorders [21]. In particular, we cannot exclude a role of this gene in the bipolar disorder presented by our proposita [22], because of its impact on the BDNF functionality. *KCNN3* overexpression [23,24] has also been related with bipolar disorder, although this correlation has not been confirmed [25].

Familiarity for bipolar disorder in the maternal line also needs to be considered, with a multifactorial contribution linked to other inherited genes and environmental factors [26].

As previously hypothesized, *LAMTOR2* intervenes in the activation of the extracellular signaling-regulated kinase and the mTOR complex 1, influencing signaling and synaptic plasticity. Consequently, it may be related with neurological diseases, anxiety, and bipolar disorder [15]. *LAMTOR2* duplication and the subsequent mTOR pathway dysregulation may thus contribute to the complex neuropsychiatric disorder observed in our patient.

Our patient had never suffered from seizures, in contrast to a patient carrying a microduplication encompassing *ADAR*, considered as a candidate gene for epilepsy [13].

White matter anomalies may be dependent on the involvement of *ADAR* itself [27], which is partially embodied. Mutation analysis of *ADAR* has been performed in order to exclude a second imbalance potentially causing recessive Aicardi–Goutieres Syndrome 6, which resulted negative. Abnormal development of the white matter has been also related to *LMNA* [28,29]. In particular, a patient with 1q22q23.1 microduplication encompassing this gene presented global developmental delay, severe muscular hypotonia, and craniosynostosis associated with brain findings, including thin and hypoplastic corpus callosum and white matter anomalies [14]. Furthermore, we cannot exclude the contribution of the mild perinatal suffering reported in our patient’s history to the leukopathy.

*MSTO1* might contribute to the mild ataxic traits described in our patient [30], while the involvement in the duplicated region of genes implied in urinary tract development (i.e., *MUC1*, *GLMP*) could explain her horseshoe kidneys [31,32,33].

## 5. Conclusions

In order to achieve a comprehensive diagnosis, the approach to complex neuropsychiatric pictures cannot overlook an integration between first-tier diagnostic tests, namely Array-CGH, and more advanced techniques (i.e., next generation sequencing), especially if ID and malformations are associated with neurological and psychiatric features. Indeed, although Array-CGH could be considered obsolete, it provides a large amount of information otherwise not detectable by other more advanced analyses. Therefore, the interpretation of Array-CGH results may be reconsidered over the years in the light of a more profound knowledge of the function of genes encompassed in chromosomal imbalances.

## Data Availability

Publicly available datasets were analyzed in this study. This data can be found here: https://decipher.sanger.ac.uk/, accessed on 28 December 2020; https://www.genome.ucsc.edu/, accessed on 28 December 2020; https://www.genecards.org/, accessed on 11 January 2021; https://omim.org/, accessed on 15 January 2021.
